# Negative Impacts of Human Land Use on Dung Beetle Functional Diversity

**DOI:** 10.1371/journal.pone.0017976

**Published:** 2011-03-23

**Authors:** Felipe Barragán, Claudia E. Moreno, Federico Escobar, Gonzalo Halffter, Dario Navarrete

**Affiliations:** 1 Centro de Investigaciones Biológicas, Universidad Autónoma del Estado de Hidalgo, Pachuca, Hidalgo, México; 2 Red de Ecoetología, Instituto de Ecología, A.C., Xalapa, Veracruz, México; 3 El Colegio de la Frontera Sur, San Cristóbal de las Casas, Chiapas, México; University of Western Ontario, Canada

## Abstract

The loss of biodiversity caused by human activity is assumed to alter ecosystem functioning. However our understanding of the magnitude of the effect of these changes on functional diversity and their impact on the dynamics of ecological processes is still limited. We analyzed the functional diversity of copro-necrophagous beetles under different conditions of land use in three Mexican biosphere reserves. In Montes Azules pastures, forest fragments and continuous rainforest were analyzed, in Los Tuxtlas rainforest fragments of different sizes were analyzed and in Barranca de Metztitlán two types of xerophile scrub with different degrees of disturbance from grazing were analyzed. We assigned dung beetle species to functional groups based on food relocation, beetle size, daily activity period and food preferences, and as measures of functional diversity we used estimates based on multivariate methods. In Montes Azules functional richness was lower in the pastures than in continuous rainforest and rainforest fragments, but fragments and continuous forest include functionally redundant species. In small rainforest fragments (<5 ha) in Los Tuxtlas, dung beetle functional richness was lower than in large rainforest fragments (>20 ha). Functional evenness and functional dispersion did not vary among habitat types or fragment size in these reserves. In contrast, in Metztitlán, functional richness and functional dispersion were different among the vegetation types, but differences were not related to the degree of disturbance by grazing. More redundant species were found in submontane than in crassicaule scrub. For the first time, a decrease in the functional diversity in communities of copro-necrophagous beetles resulting from changes in land use is documented, the potential implications for ecosystem functioning are discussed and a series of variables that could improve the evaluation of functional diversity for this biological group is proposed.

## Introduction

One of the main challenges in ecology is understanding how habitat alteration affects biodiversity. Species richness is most commonly used to evaluate the impact, but assumes that all species contribute equally to the functioning of the ecosystem. This is why when evaluating biodiversity, complementary information—such as the diversity of the ecological roles of the species [1]–[5]—should be included. Though there are few studies that evaluate the influence of anthropic changes on functional diversity under field conditions (most of the evidence comes from experiments carried out under controlled conditions), it has been proposed that human activities result in the loss or addition of species with certain functional traits and therefore modify the functioning of ecosystems [6]. For this reason, and given the worrying rate of habitat transformation, it is imperative to analyze changes in biodiversity under different types of land use with complementary approaches. Protected natural areas are at the core of local and global conservation efforts, but these contrast with the surrounding areas modified by humans given that in many regions they are surrounded by areas where agricultural crops are grown and livestock is raised [7]. These regions therefore offer ideal systems for evaluating the impact of human activities.

Functional diversity is a component of biodiversity and expresses the degree of functional differences among species (*i.e.*, the way in which they use resources). Even though functional diversity affects the integrity of ecological processes and ecosystem dynamics [1], [3], there is no simple, direct way of measuring it. It can however be quantified as the number of trophic levels, functional groups, life cycles, and by the resources used by species [3], [4], or using multivariate methods that summarize the functional variability in the group of species being analyzed [8]–[11]. In this study, to estimate functional diversity we use approaches based on multivariate methods: a) functional richness, measured as the total length of the branches in a functional dendrogram [8], as an analog for the measure of phylogenetic diversity proposed by Faith [12]. The latter has been used recently to evaluate the impact of habitat fragmentation on evolutionary diversity [13]; b) functional richness, measured as the amount of functional space filled by the community [10]; c) functional evenness, the regularity with which the functional space is filled by species, weighted by their abundance [10]; and d) functional dispersion, the mean distance of individual species to the centroid of all species in the community [14]. Functional diversity estimates are useful for assessing the degree of the complementarity of the characteristics or attributes among species and of the functional variation in the species of a community. They are also useful for comparing different ecological (*e.g*., type of land use) and evolutionary scenarios (*e.g*., biogeographic regions), under the assumption that changes in species richness and identity are reflected in the values of functional diversity [8], [11].

Our analysis focuses on the functional diversity of beetles belonging to subfamily Scarabaeinae (Coleoptera: Scarabaeidae), known for their role in ecosystem functioning owing to their dependence on vertebrate dung, particularly that of mammals, as a food source and for reproduction [15]. Dung beetles have recently received increasing attention as indicators of changes in land use [16] and the health status of pastures [17]. The activities of these beetles are linked to a wide variety of ecological processes including breaking down and moving excrement, the incorporation of organic matter into the soil, bioturbation (*i.e.*, moving and mixing soil particles), controlling the parasites and flies that affect livestock, pets and people, and secondary seed dispersal, see [18] and references cited therein. The vegetation structure, as well as the spatial and temporal availability of dung in a given habitat modulates the dung beetle assemblage [19]. The intensification of agriculture and increased livestock density in tropical and subtropical regions are also known to affect the dung beetle community [20], though there is still no information about the consequences of these changes to functional diversity.

Based on the idea that changes caused by people affect species richness and composition in dung beetles, we expect that some functional groups will be more sensitive than others to changes in land use, and that this will be detected as a decrease in functional diversity (including functional richness, functional evenness, and functional dispersion) in deteriorated habitats. We also expect that functional groups with large species will be those most affected by habitat loss, because they require ample home ranges to survive, making them more vulnerable to extinction [21].

## Methods

### Study sites and beetle sampling

Data for three biosphere reserves from central and southeastern Mexico were used: Montes Azules, Los Tuxtlas and Barranca de Metztitlán. These reserves were selected because their communities of dung beetles have been sufficienty sampled. In addition, these three reserves offer contrasting ecological (land use) and biogeographical scenarios, allowing us to evaluate any changes in functional diversity using the same taxonomic group.

#### The Montes Azules Biosphere Reserve

With an area of 332 thousand hectares, this reserve is located on the eastern side of the state of Chiapas (16°05′–16°20′N; 90°42′–91°08′W) in the region known as the Lacandona Rainforest. The climate is warm-humid with rainfall in the summer (>3000 mm) and a mean annual temperature >22°C [22]. Altitude is 100 to 900 m a.s.l. and the reserve is mainly covered by tall rainforest. On a regional scale 63% of the original vegetation has been transformed for agricultural use where the slash-and-burn technique is used to grow beans, corn and grass for cattle. Only 37% of the original vegetation remains, most of which is found within the reserve [23].

Sampling was done between October 2003 and August 2004 at 38 sites located throughout three habitats: well preserved rainforest (14 sites), rainforest fragments (14 sites) and open pastures used for cattle (10 sites) [24]. Ten pitfall traps were used per site, separated by 30 m along a transect. The minimum distance between sites was 630 m and the maximum distance was 18 km. Traps were alternately baited with human excrement (five) and rotting fish (five), and were left open for 48 h, see [24] for more detail.

#### The Los Tuxtlas Biosphere Reserve

Located in the state of Veracruz on the Gulf of Mexico coast (18°8′–18°45′N; 94°37′–95°22′W), this reserve has an area of 155 122 ha and rises from sea level at the coast to 1700 m a.s.l. (Sierra de Santa Marta Mountain Range) [25]. The climate is warm, with a mean annual temperature <20°C and a mean annual precipitation of 4500 mm. Rainfall is markedly seasonal with a rainy season that lasts from June to February and a dry season from March to May. The dominant vegetation type below 700 m a.s.l. is tropical rainforest [26]. There has been a notable decrease in the area of the rainforest over the last few decades. It has been estimated that by 1980 the area that had been converted into cattle pasture was close to 75% of the original area covered by rainforest [27]. Currently, the remaining rainforest is in fragments of different sizes which together represent 15% of the total area [28].

Sampling was done between June and August 2003 in 30 rainforest fragments of different sizes (range: 1.3 to 244 ha) in the northern part of the reserve (Balzapote Municipality; Escobar, unpublished data) between 100 and 350 m a.s.l. A linear transect was set up in each fragment with 10 pitfall traps, five baited with human excrement and five with carrion. The traps were separated by 50 m and left open for 48 h. For this study, the rainforest fragments were classified according to the criteria of Arroyo-Rodriguez et al. [29]: small (<5 ha), medium-sized (5–20 ha) and large (>20 ha).

#### The Barranca de Metztitlán Biosphere Reserve

Located in the state of Hidalgo (20°14′–20°45′N; 98°23′–98°57′W) this reserve covers 96 thousand ha. The climate is hot and dry, with 413.9 mm annual precipitation and a mean annual temperature of 21°C [30]. The landscape is characterized by a wide diversity of semi-arid vegetation types, with the notable presence of submontane and crassicaule scrub [31],[32] between 1300 and 1800 m a.s.l.

Raising livestock is one of the main sources of income for the inhabitants of the region so there are herds of sheep and goats that graze throughout the area, along with freely ranging cattle and horses. Pressure on the ecosystems is, therefore, notable particularly on the submontane scrub where livestock activity is more intense, while in the crassicaule scrub the extraction of different species of cactus is the main cause of deterioration [32].

Sampling was done in two types of vegetation: crassicaule scrub and submontane scrub [33]. Two areas with different degrees of disturbance were selected in each type of vegetation: one with a lot of livestock activity and a marked decrease in plant cover (which we refer to as open), and the other with less disturbance (closed). Six sampling sites were set up in each area (24 sites in total), separated by 500 m. At each site four pitfall traps were set, separated by 50 m and baited with a mixture (3∶1) of sheep and horse dung. Carrion—which is usually used as a complementary bait when doing beetle inventories—was not used in this sampling owing to its low capture effectiveness in this type of environment. Sampling was done six times between June and September 2006 and the traps were active for six consecutive days each time see [33] for further details.

### Functional characteristics of dung beetles

Four characteristics are traditionally used, alone or in combination, to identify the functional groups or guilds of species that make up the communities of beetles belonging to subfamily Scarabaeinae, given that each trait has a particular impact on the functions of the ecosystem.

The first is related to food relocation and there are three categories: in the first, beetles arrive at the dung and shape a ball which they roll for a certain distance and then bury, or very occasionally leave on the soil surface of the soil. These are called telecoprids or rollers. In the second group the beetles bury portions of dung in tunnels that extend straight downwards or at an oblique angle to the site where the dung was originally deposited. These are called paracoprids or tunnelers. In the third group, the beetles live and nest inside the dung and are known as endocoprids [15], [19].

The second characteristic is the size of the beetle. Total length is usually used and small species are <10 mm (though this varies depending on the study and may be as much as 13 mm) and large species are >10 mm. This arbitrary classification has been used in previous studies with this group, *e.g.* [19], [24], [34] and was the only way to incorporate size in this work given the information available. Ideally, size would be incorporated as a continuous variable of several body lengths, or as biomass.

Other studies classify the beetles based on a third characteristic depending on the time of day when they are active. Diurnal beetles are active after sunrise and before sundown, and nocturnal beetles are active during the night.

The final characteristic we used is diet. Coprophagous species are those that have a strong affinity for dung, necrophagous species are those that prefer carrion, generalists will eat either and there is a recently defined category, trophic specialists, for beetles that eat fruit or fungi [35].

### Data analysis

In this study we define the sampling sites within each reserve as independent samples of the dung beetle community. In order to standardize the analyses, we evaluated the degree of completeness of the inventories for each site as the percentage of observed species with respect to the number of species predicted by Chao1, a nonparametric estimator of species richness based on species abundance that takes the rare species in the sample (≤2 individuals) into account [36]. For the analyses, data was only used for those sites with an inventory completeness ≥80%.

To classify the species qualitatively by functional group and obtain the quantitative value of functional diversity, a presence absence matrix of functional traits was generated for each species, in each of the three reserves. Information on functional traits was obtained from the literature and corroborated by experts. The traits used were food relocation behavior (telecoprid, paracoprid, endocoprid), size (small, large), activity (diurnal, nocturnal) and food preference (coprophage, necrophage, generalist, trophic specialist) (Table S1).

This information was used to calculate four estimates of functional diversity. First, we use an estimate of functional richness based on dendrogram length, FR_D_ [8]. This estimate was selected because it was the first index available for multivariate data [8] and has been used in some empirical studies, *e.g.* [37]. For calculating this index we used the routine written by O. L. Petchey for the statistical program R [38]. Given that FR_D_ has been found to be strongly correlated with species richness [11], we choose a second estimate of functional richness based on the volume of a multidimensional functional space, FR_V_, measured as a convex hull volume [10]. These two estimates of functional richness are based on different algorithms and thus, they could have differential responses, so we decided to include both of them. As a third estimate we computed the functional evenness, FEve, as the regularity with which the functional space is filled by species, using the regularity of branch lengths in a minimum spanning tree and evenness in species abundances [10]. Finally, the fourth estimate was functional dispersion, FDis, measured as the mean distance of individual species to the centroid of all species in the community, where the weights are species' relative abundances [14]. The last three estimates were calculated using the FD package [14] for the R program [38], which allows the inclusion of any number of traits, and different trait types.

The values of FR_D_, FR_V_, FEve and FDis between groups of habitats within each reserve were compared using one-way ANOVAs when data were normally distributed, and with a Kruskal-Wallis test when the data failed normality tests. Post hoc paired Tukey tests were performed. In Montes Azules there were three types of habitat (continuous rainforest, rainforest fragments, pastures), in Los Tuxtlas there were three sizes of rainforest fragment (small, medium and large), and in Metztitlán there were four types of habitat (open crassicaule scrub, closed crassicaule scrub, open submontane scrub and closed submontane scrub).

## Results

### Montes Azules

**Figure 1:**
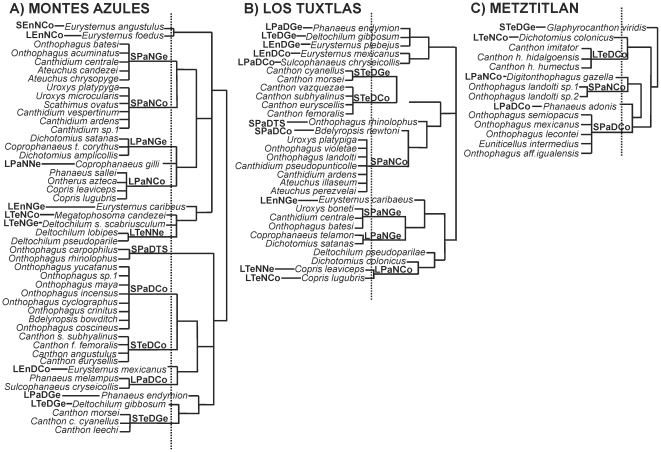
Dendrogram of the species forming functional groups. Functional groups were considered at an arbitrary Euclidian distance of 1.5 (dotted line). The branch length of these dendrograms was used to analyze dung beetle functional diversity (FD) in (A) the Montes Azules Biosphere Reserve, (B) the Los Tuxtlas Biosphere Reserve, and (C) the Barranca de Metztitlán Biosphere Reserve. The names of the functional groups are combinations of the following characteristics: S = small, L = Large, Pa = paracoprid, En = endocoprid, Te = telecoprid, D = diurnal, N = nocturnal, Ge = generalist, Co = coprophage, Ne = necrophage, and TS = trophic specialist.

**Figure 2:**
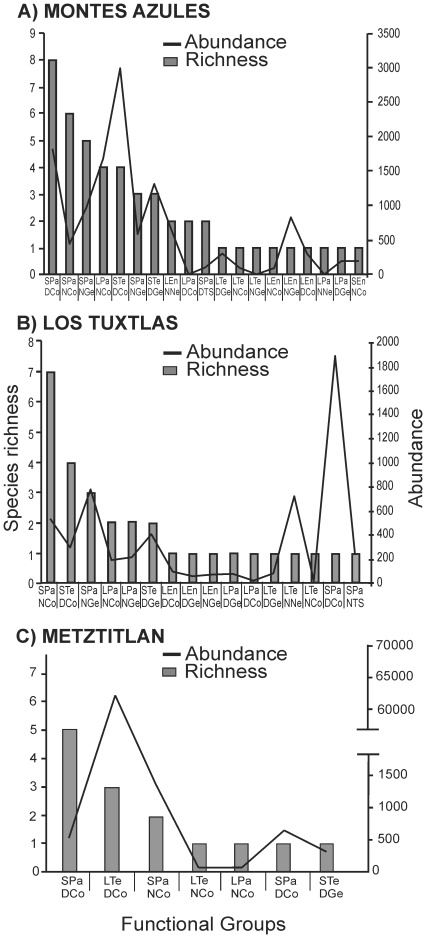
Species richness and number of dung beetles per functional group. The figure shows the communities from (A) Montes Azules, (B) Los Tuxtlas and (C) Barranca de Metztitlán. The names of the functional groups are combinations of the following characteristics: S = small, L = Large, Pa = paracoprid, En = endocoprid, Te = telecoprid, D = diurnal, N = nocturnal, Ge = generalist, Co = coprophage, Ne = necrophage, and TS = trophic specialist.

**Figure 3:**
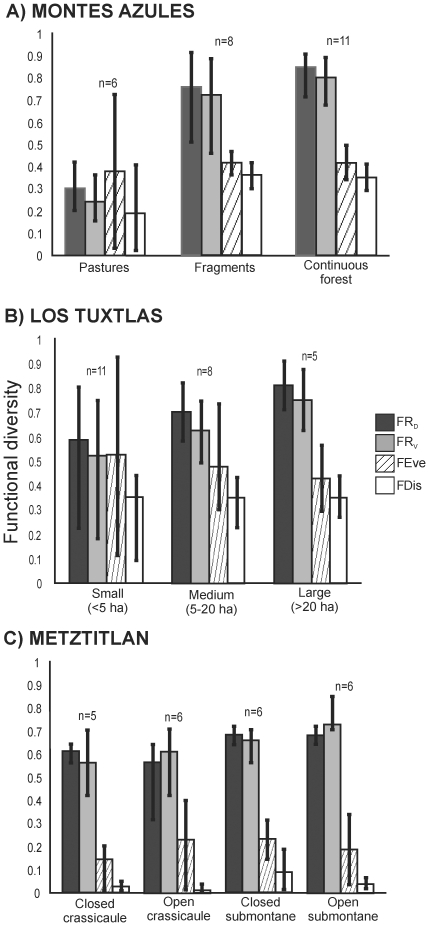
Mean values of functional diversity for the dung beetle communities under different habitat conditions. (A) Montes Azules, (B) Los Tuxtlas and (C) Barranca de Metztitlán. Error bars are standard error. The value of FR_D_ (functional richness) is based on dendrogram length, FR_V_ (funcional richness) is a convex hull volume of functional space, FEve (functional evenness) is the regularity with wich the functional space is filled by species, weighteg by their abundance, and FDis (functional dispersion) is the mean distance of individual species to the centroid of all species in the community.

Twenty-five of the 38 (65.79%) sites studied in Montes Azules had a complete inventory (≥80% complete): 11 in continuous rainforest, eight in rainforest fragments and six in pastures. These 25 communities had 48 species of dung beetle belonging to 19 functional groups (Figure 1A). The functional group with the most individuals was small, telecoprid, diurnal coprophages (STeDCo), while species richness was highest for small, paracoprid, diurnal coprophages (SPaDCo) (Figure 2A). FR_D_ and FR_V_ varied among types of habitat (*F* = 70.45 and *F* = 61.17, respectively, *P*<0.001 and df = 24 for both). The highest values were recorded for communities in the continuous rainforest, and the lowest were recorded for the pastures with both estimates (Figure 3A). There was a significant difference in mean functional richness for the communities in the pasture and the continuous rainforest (*Q* = 16.36, *P*<0.001 for FR_D_; *Q* = 14.89, *P*<0.001 for FR_V_), and between the communities of the pasture and rainforest fragments (*Q* = 12.92, *P*<0.001 for FR_D_; *Q* = 12.86, *P*<0.001 for FR_V_), but not between those of the continuous rainforest and rainforest fragments (*Q* = 2.85, *P* = 0.13 for FR_D_; *Q* = 1.31, *P* = 0.63 for FR_V_). Contrary to these trends in functional richness, the estimates of functional evenness and functional dispersion (FEve and FDis; Figure 3A) did not vary among habitat types (*H* = 0.109, *P* = 0.95; and *H* = 5.32, *P* = 0.07, respectively).

All of the 19 functional groups at Montes Azules were found in continuous forest, where the most abundant functional group was that of small, telecoprid, diurnal, coprophagous species (STeDCo), and the least abundant functional group was that of large, paracoprid, nocturnal, necrophagous species (LPaNNe). In forest fragments we only recorded 17 functional groups (two less than in continuous forests). One of the missing functional groups is LPaNNe (the least abundant in continuous forests). The most abundant functional group in these forest fragments was the small, paracoprid, diurnal, coprophagous species (SPaDCo), while the least abundant were large, telecoprids, nocturnal, generalist species (LTeNGe). In pastures only nine functional groups were found, with the small, telecoprid, diurnal generalists (STeDGe) the group most commonly associated with this habitat and the large, paracoprid, diurnal, generalists (LPaDGe) the group less abundant. The LTeNGe, which is the least abundant functional group in the fragmented forests, is one of the 10 functional groups absent in pastures. In general, large and paracoprid species are most strongly affected by habitat transformation on this reserve as it is the functional group associated with the sites of continuous rainforest.

**Figure 4:**
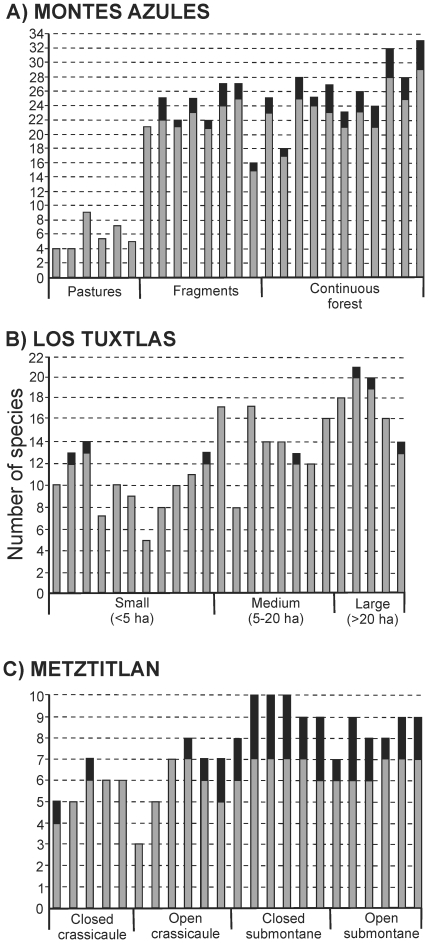
Functionally singular and redundant species in each local community. The complete bar indicates the total number of species in each community, the white segment corresponds to the functionally singular species and the black segment to the redundant species. (A) Montes Azules, (B) Los Tuxtlas and (C) Barranca de Metztitlán.

Of the 12 species found in pastures, two small, diurnal species were exclusive to this environment: *Canthon leechi* and *Onthophagus cyclographus*. The first is a telecoprid generalist and the second, a paracoprid coprophage. Another interesting result is that in pastures, all the species found are functionally different (Figure 4A), while in forest fragments and in continuous forests we found up to four redundant species in each sample, which corresponds to up to 14.81% of the total number of species per sample.

### Los Tuxtlas

Inventory was complete in 24 of the 30 forest fragments (80%) sampled in Los Tuxtlas: 11 in small fragments, eight in medium-sized fragments and five in large fragments. In these 24 fragments there were 30 species in 16 functional groups (Figure 1B). The functional group with the most individuals was the small, paracoprid, diurnal coprophages (SPaDCo) while species richness was greatest for the small, paracoprid, nocturnal coprophages (SPaNCo) (Figure 2B). Mean functional richness varied with fragment size (*F* = 4.85, *P* = 0.018, df = 23 for FR_D_; *F* = 5.98, *P* = 0.017, df = 23 for FR_V_). Functional richness, with both FR_D_ and FR_V_, was highest in the communities of large fragments, followed by those of medium-sized fragments, and was lowest in the communities inhabiting the small fragments (Figure 3B). There was a significant difference in mean functional richness for the communities in small and large fragments (*Q* = 4.27, *P* = 0.017 for FR_D_; *Q* = 4.40, *P* = 0.014 for FR_V_), but not for the communities in small and medium-sized fragments (*Q* = 2.55, *P* = 0.192 for FR_D_; *Q* = 2.29, *P* = 0.257 for FR_V_), or for those in medium-sized and large fragments (*Q* = 1.96 *P* = 0.366 for FR_D_; *Q* = 2.29, *P* = 0.26 for FR_V_). Functional evenness and functional dispersion (FEve and FDis) did not vary among types of habitat (Figure 3B; *F* = 0.51, *P* = 0.61; and *F* = 0.0089, *P* = 0.99, respectively).

Fifteen of the total 16 functional groups were recorded in the large fragments of Los Tuxtlas. The dominant functional group in large, medium-sized and small fragments was that of small, paracoprid, diurnal coprophages (SPaDCo). The least abundant group in large forest fragments was large, paracoprid, diurnal and coprophage species (LPaDCo). This functional group was not found in medium-sized fragments, where only 14 functional groups were recorded. In these medium-sized fragments the least abundant functional group was that of large, telecoprid, nocturnal, coprophagous species (LTeNCo), which is absent in small fragments. In the small fragments, the number of functional groups was 12 (of the 16 for this reserve).

At Los Tuxtlas 22 of the 30 species collected were found in the small fragments, and only two species were exclusive to these small fragments: *Onthophagus landolti* and *Onthophagus violetae*, both of which are small, paracoprid, nocturnal, coprophages (SPaNCo). In all of the fragments, the small species are more abundant than the large ones, and paracoprid species dominate, though species that relocate their food this way were more abundant in the small fragments and their proportion decreased as fragment size increased. There is no pattern in the presence of functionally singular species with respect to fragment size, and only one species was detected as redundant in some samples (Figure 4B).

### Barranca de Metztitlán

In Metztitlán 23 of the 24 sites (98.5%) had complete inventories: five in closed crassicaule scrub, six in open crassicaule scrub, six in closed submontane scrub and six in open submontane scrub. These 23 communities were home to 14 species of Scarabaeinae that belonged to seven functional groups (Figure 1C). The most abundant functional group was the large, telecoprid, diurnal coprophages (LTeDCo), while the group with the most species was small, paracoprid, diurnal coprophages (SPaDCo) (Figure 2C), owing to the marked dominance of *Canthon humectus hidalgoensis* [33]. There was no significant difference in mean functional richness among the four types of habitat using the FR_D_ (*H* = 4.65, *P* = 0.19), but significant differences were found using the FR_V_ estimate (*F* = 3.506, *P* = 0.035, df = 20) and only the values for closed submontane scrub and open crassicaule scrub were statistically different (*Q* = 4.33, *P* = 0.03), the other combinations were not (P>0.10). Functional evenness was not significantly different among habitat types (*H* = 2.59, *P* = 0.46), but followed the same trend as FR_V_, functional dispersion was different (*H* = 9.85, *P* = 0.02). Only the values for closed submontane scrub and open crassicaule scrub were statistically different (*Q* = 2.93, *P*<0.05); the other combinations were not (*P*>0.05).

In Metztitlán, submontane scrub had all seven functional groups, while crassicaule scrub had six. All sites were dominated by large, telecoprid, diurnal coprophages (LTeDCo) because of the high abundance of *Canthon humectus hidalgoensis* and in general, the less abundant groups were those including large paracoprids. In crassicaule scrub five of the 11 samples included one of two redundant species, while in the other six samples of this habitat all the species are functionally different (Figure 4C). However, in the submontane scrub all the samples included redundant species (up to 3 species, which represent up to 33.33% of the species richness per sample).

## Discussion

Several studies have documented the impact of anthropogenic changes in land use on biodiversity, using species richness as the point of comparison, *e.g.* [33], [35], [39]. For dung beetles, in addition to a decrease in richness, a decrease in abundance has been observed, along with changes in species composition that depend on the degree of habitat transformation, see [20] and references cited therein. Even so, the impact of this type of transformation has not been evaluated from a functional perspective. In this study we document for the first time the drastic decrease in the functional diversity of dung beetle communities that results from habitat alteration in two of the three biosphere reserves studied. This could have serious implications for the dynamics of the ecological processes regulated by this group of insects inside these protected areas.

In Montes Azules the highest functional richness values were recorded for dung beetle communities in continuous rainforest and in rainforest fragments, while in pasture communities a loss of functional diversity was evident. In Los Tuxtlas, small fragments were seen to have low functional richness values compared to medium-sized and large fragments. It is clear that the changes in functional diversity among habitat types in these reserves are due to variation in functional richness, which was detected by the two richness estimators used in this study (FR_D_ and FR_V_). In theory, when the number of species increases, one way that local communities can change is by increasing the volume of the niche space to accommodate the new species (the “volume-increasing assembly mechanism”) [40], [41]. This mechanism may be regulating dung beetle communities at Montes Azules and Los Tuxtlas, when the niche volume (FR_V_) increases and the interspecific distance (FEve and FDis) remains unaltered from the simplified communities of pastures to continuous rainforest sites at Montes Azules, and from the small to large fragments in Los Tuxtlas. Although the implications of the loss and fragmentation of habitat for the dynamics of the ecological processes regulated by the dung beetles are as yet unknown, in the central Amazon Klein [42] observed a marked decrease in the rate of dung removal correlated with the decrease in dung beetle species richness owing to fragmentation. In the Colombian Andes Giraldo [43] found that greater dung removal was associated with an increase in the abundance of large dung beetle species.

In contrast, in a different ecological setting with a different history, where disturbance is mainly caused by grazing, we only found differences for one of the two estimates of functional richness and for functional dispersion between the least similar environments (closed submontane and open crassicaule scrub), so there was no evidence of a marked impact on the functional diversity of dung beetle communities in the xerophile scrub of the Barranca de Metztitlán reserve. One explanation is that Meztitlán is in a semi-arid region of the Mexican High Plateau populated by species that are able to use open areas that are devoid of vegetation and can take advantage of the additional dung made available by livestock. Furthermore, unlike the lowlands with tropical rainforest, in the Barranca de Metztitlán it is not possible to identify the vast, completely transformed areas that are used for livestock because the animals are allowed to range freely throughout the reserve. Our results support the proposal of Escobar [44] who suggests that the impact of the livestock has a differential effect depending on the biogeographic and ecological characteristics of the beetle community that inhabits each region.

In spite of the biogeographic and climate differences between reserves, beetles that are small, paracoprid coprophages (SPaDCo and SPaNCo) comprised the functional groups with the most species. Even so, this was not the case for abundance (see Figure 2). It is known that species abundance can be more important than species richness [45], [46]. At present, however, the relative contribution of species richness, abundance or biomass to ecological function remains an area of uncertainty within the biodiversity, ecosystem, and functional lines of research. The differences in the dominance of the functional groups in terms of richness and abundance according to the ecological (land use) and biogeographic context (historical) should also be analyzed taking into account species biomass when this information is available.

In Montes Azules the large, paracoprid, nocturnal coprophages (LPaNCo) were the most sensitive to habitat transformation. This result coincides with that described by Larsen [21], who reports that the species that are susceptible to extinction owing to fragmentation are large, specialists that live in the forest, or are rare. The disappearance of large species has a big impact on several ecological processes because these are the beetles that remove the most dung and do so most quickly. According to Slade [47], the disappearance of large paracoprids reduced dung removal by up to 75% and this could have a large impact on nutrient recycling and secondary seed dispersal. These processes could be affected in areas with pasture, although the impact of a mechanism that compensates for the disappearance of large species with the high abundance of small-sized species has yet to be evaluated in functional terms. Another important pattern found in our study is that the few species that inhabit pastures are functionally singular, while in fragments and continuous forests there are almost always some redundant species. However, the presence of redundant species may be an artifact of the coarse measure of species traits applied in this study. The incorporation of quantitative traits directly linked to ecosystem functioning, such as dung removal rates, may shed light on the real existence of redundant species and the relationship between biodiversity and ecosystem processes.

The results we present should be interpreted with caution given that they only represent a single line of evidence regarding the magnitude of the changes in functional diversity resulting from human activities. They do however, form the appropriate basis for new questions regarding how the rate of functional processes changes when the richness and abundance of functional groups change, and about the relationship between these changes and increased intensity of soil use. This information will doubtless contribute to consolidating one of the premises of modern conservation: that of managing biodiversity properly to ensure the continued availability of the services provided by the ecosystems that directly and indirectly affect human well-being.

## Supporting Information

Table S1 List of species used for the analysis of functional diversity in each reserve (MAZ  =  Montes Azules, LTX  = Los Tuxtlas, ME  =  Metztitlán) with the functional characteristics that are traditionally used to assign species to a guild.
(DOCX)
