# Supplementary material for: Negative Impacts of Human Land Use on Dung Beetle Functional Diversity
Source: PLoS One. 2011 Mar 23;6(3):e17976. doi: 10.1371/journal.pone.0017976 (PMC3063245; doi:10.1371/journal.pone.0017976)
Supplement: Table S1 — List of species used for the analysis of functional diversity in each reserve (MAZ = Montes Azules, LTX = Los Tuxtlas, ME = Metztitlán) with the functional characteristics that are traditionally used to assign species to a guild. (DOCX) [file pone.0017976.s001.docx]

| **Species** | **Reserve** | **Size** |  | **Food Relocation** | **Activity Period** | **Diet** |
| --- | --- | --- | --- | --- | --- | --- |
|  |  |  |  |  |  |  |
| *Ateuchus candezei (Harold, 1868)* | MAZ | Small |  | Paracoprid | Nocturnal | Generalist |
| *Ateuchus chrysopyge (Bates, 1887)* | MAZ | Small |  | Paracoprid | Nocturnal | Generalist |
| *Ateuchus illaseum (Harold, 1868)* | LTX | Small |  | Paracoprid | Nocturnal | Coprophage |
| *Ateuchus perezvelai (Kohlmann, 2000)* | LTX | Small |  | Paracoprid | Nocturnal | Coprophage |
| *Bdelyropsis bowditchi (Paulian, 1939)* | MAZ | Small |  | Paracoprid | Diurnal | Coprophage |
| *Bdelyropsis newtoni (Howden, 1971)* | LTX | Small |  | Paracoprid | Diurnal | Coprophage |
| *Canthidium ardens (Bates, 1887)* | LTX, MAZ | Small |  | Paracoprid | Nocturnal | Coprophage |
| *Canthidium centrale (Boucomont, 1928)* | LTX, MAZ | Small |  | Paracoprid | Nocturnal | Generalist |
| *Canthidium pseudopunticolle (Howden & Young, 1981)* | LTX | Small |  | Paracoprid | Nocturnal | Coprophage |
| *Canthidium sp1.* | MAZ | Small |  | Paracoprid | Nocturnal | Coprophage |
| *Canthidium vespertinum (Howden & Young, 1981)* | MAZ | Small |  | Paracoprid | Nocturnal | Coprophage |
| *Canthon angustatus (Harold, 1868)* | MAZ | Small |  | Telecoprid | Diurnal | Coprophage |
| *Canthon cyanellus cyanellus (LeConte, 1859)* | LTX, MAZ | Small |  | Telecoprid | Diurnal | Generalist |
| *Canthon euryscellis (Bates, 1887)* | LTX, MAZ | Small |  | Telecoprid | Diurnal | Coprophage |
| *Canthon femoralis femoralis (Chevrolat, 1834)* | LTX, MAZ | Small |  | Telecoprid | Diurnal | Coprophage |
| *Canthon humectus hidalgoensis (Bates, 1887)* | MET | Large |  | Telecoprid | Diurnal | Coprophage |
| *Canthon humectus humectus (Say, 1832)* | MET | Large |  | Telecoprid | Diurnal | Coprophage |
| *Canthon imitator (Brown, 1946)* | MET | Large |  | Telecoprid | Diurnal | Coprophage |
| *Canthon leechi (Martínez, Halffter & Halffter, 1964)* | MAZ | Small |  | Telecoprid | Diurnal | Generalist |
| *Canthon morsei (Howden, 1966)* | LTX, MAZ | Small |  | Telecoprid | Diurnal | Generalist |
| *Canthon subhyalinus subhyalinus (Harold, 1867)* | LTX, MAZ | Small |  | Telecoprid | Diurnal | Coprophage |
| *Canthon vazquezae (Martínez, Halffter & Halffter, 1964)* | LTX | Small |  | Telecoprid | Diurnal | Coprophage |
| *Copris laeviceps (Harold, 1869)* | LTX, MAZ | Large |  | Paracoprid | Nocturnal | Coprophage |
| *Copris lugubris (Boheman, 1858)* | LTX, MAZ | Large |  | Paracoprid | Nocturnal | Coprophage |
| *Coprophanaeus gilli (Arnaud, 1997)* | MAZ | Large |  | Paracoprid | Nocturnal | Necrophage |
| *Coprophanaeus telamon corythus (Harold, 1863)* | LTX, MAZ | Large |  | Paracoprid | Nocturnal | Generalist |
| *Deltochilum gibbosum sublaeve (Bates, 1887)* | LTX, MAZ | Large |  | Telecoprid | Diurnal | Generalist |
| *Deltochilum lobipes (Bates, 1887)* | MAZ | Large |  | Telecoprid | Nocturnal | Necrophage |
| *Deltochilum pseudoparilae (Paulian, 1938)* | LTX, MAZ | Large |  | Telecoprid | Nocturnal | Necrophage |
| *Deltochilum scabriusculum scabriusculum (Bates, 1887)* | MAZ | Large |  | Telecoprid | Nocturnal | Generalist |
| *Dichotomius amplicollis (Harold, 1869)* | MAZ | Large |  | Paracoprid | Nocturnal | Generalist |
| *Dichotomius colonicus (Say, 1835)* | LTX, MET | Large |  | Telecoprid | Nocturnal | Coprophage |
| *Dichotomius satanas (Harold, 1867)* | LTX, MAZ | Large |  | Paracoprid | Nocturnal | Generalist |
| *Digitonthophagus gazella (Fabricius, 1787)* | MET | Large |  | Paracoprid | Nocturnal | Coprophage |
| *Euoniticellus intermedius (Reiche, 1849)* | MET | Small |  | Paracoprid | Diurnal | Coprophage |
| *Eurysternus angustulus (Harold, 1869)* | MAZ | Small |  | Endocoprid | Nocturnal | Coprophage |
| *Eurysternus caribaeus (Herbst, 1789)* | LTX, MAZ | Large |  | Endocoprid | Nocturnal | Generalist |
| *Eurysternus foedus (Guérin-M. 1844)* | MAZ | Large |  | Endocoprid | Nocturnal | Coprophage |
| *Eurysternus mexicanus (Harold, 1869)* | LTX, MAZ | Large |  | Endocoprid | Diurnal | Coprophage |
| *Eurysternus plebejus (Harold, 1980)* | LTX | Large |  | Endocoprid | Diurnal | Generalist |
| *Glaphyrocanthon viridis (de Beauvois, 1805)* | MET | Small |  | Telecoprid | Diurnal | Generalist |
| *Megathoposoma candezei (Harold, 1876)* | MAZ | Large |  | Telecoprid | Nocturnal | Coprophage |
| *Ontherus azteca (Harold, 1869)* | MAZ | Large |  | Paracoprid | Nocturnal | Coprophage |
| *Onthophagus acuminatus (Harold, 1880)* | MAZ | Small |  | Paracoprid | Nocturnal | Generalist |
| *Onthophagus aff. Igualensis (Bates, 1887)* | MET | Small |  | Paracoprid | Diurnal | Coprophage |
| *Onthophagus aff.landolti (Harold, 1980)1* | MET | Small |  | Paracoprid | Noctural | Coprophage |
| *Onthophagus aff.landolti (Harold, 1980)2* | MET | Small |  | Paracoprid | Nocturnal | Coprophage |
| *Onthophagus batesi (Howden & Critwright, 1963)* | LTX, MAZ | Small |  | Paracoprid | Nocturnal | Generalist |
| *Onthophagus carpophilus (Pereira & Halffter, 1961)* | MAZ | Small |  | Paracoprid | Diurnal | Trophic specialist |
| *Onthophagus coscineus (Bates, 1887)* | MAZ | Small |  | Paracoprid | Diurnal | Coprophage |
| *Onthophagus crinitus (Harold, 1869)* | MAZ | Small |  | Paracoprid | Diurnal | Coprophage |
| *Onthophagus cyclographus (Bates, 1887)* | MAZ | Small |  | Paracoprid | Diurnal | Coprophage |
| *Onthophagus incensus (Say, 1835)* | MAZ | Small |  | Paracoprid | Diurnal | Coprophage |
| *Onthophagus landolti (Harold, 1880)* | LTX | Small |  | Paracoprid | Nocturnal | Coprophage |
| *Onthophagus lecontei (Harold, 1871)* | MET | Small |  | Paracoprid | Diurnal | Coprophage |
| *Onthophagus maya (Zunino, 1981)* | MAZ | Small |  | Paracoprid | Diurnal | Coprophage |
| *Onthophagus mexicanus (Bates, 1887)* | MET | Small |  | Paracoprid | Diurnal | Coprophage |
| *Onthophagus rhinolophus (Harold, 1869)* | LTX, MAZ | Small |  | Paracoprid | Diurnal | Trophic specialist |
| *Onthophagus semiopacus (Harold, 1869)* | MET | Small |  | Paracoprid | Diurnal | Coprophage |
| *Onthophagus sp1.* | MAZ | Small |  | Paracoprid | Diurnal | Coprophage |
| *Onthophagus violetae (Zunino & Halffter, 1997)* | LTX | Small |  | Paracoprid | Nocturnal | Coprophage |
| *Onthophagus yucatanus (Delgado, 2006)* | MAZ | Small |  | Paracoprid | Diurnal | Coprophage |
| *Phanaeus adonis (Harold, 1863)* | MET | Large |  | Paracoprid | Diurnal | Coprophage |
| *Phanaeus endymion (Harold, 1863)* | LTX, MAZ | Large |  | Paracoprid | Diurnal | Generalist |
| *Phanaeus melampus (Harold, 1863)* | MAZ | Large |  | Paracoprid | Diurnal | Coprophage |
| *Phanaeus sallei (Harold, 1863)* | MAZ | Large |  | Paracoprid | Nocturnal | Coprophage |
| *Scatimus ovatus (Harold, 1862)* | MAZ | Small |  | Paracoprid | Nocturnal | Coprophage |
| *Sulcophanaeus chryseicollis (Harold, 1863)* | LTX, MAZ | Large |  | Paracoprid | Diurnal | Coprophage |
| *Uroxys boneti (Pereira & Halffter, 1961)* | LTX | Small |  | Paracoprid | Nocturnal | Generalist |
| *Uroxys microcularis (Howden & Young, 1981)* | MAZ | Small |  | Paracoprid | Nocturnal | Coprophage |
| *Uroxys platypiga (Howden & Young, 1981)* | MAZ, LTX | Small |  | Paracoprid | Nocturnal | Coprófaga |
